# New and Emerging Research Models for Sepsis

**DOI:** 10.3390/cells15040312

**Published:** 2026-02-07

**Authors:** Saichaitanya Nallajennugari, Xiang Li, Mingui Fu

**Affiliations:** Department of Biomedical Science, Shock/Trauma Research Center, School of Medicine, University of Missouri Kansas City, Kansas City, MO 64108, USA; srnnkz@umkc.edu (S.N.); xlkgk@umkc.edu (X.L.)

**Keywords:** sepsis, animal models, infection, immune response, organ-on-chip

## Abstract

Human sepsis is a complex disease that manifests with a diverse range of phenotypes and inherent variability among individuals, making it hard to develop a comprehensive animal model. Despite this difficulty, numerous animal models have been developed that capture many key aspects of human sepsis. Though the animal models have contributed to the fundamental advances in understanding the pathogenesis of septic patients, the translational value of these models has been constantly questioned because many clinical trials of targeted therapies based on the advances in animal models have failed, highlighting the urgent need for developing new research models or refining previous animal models for sepsis research. In this review, we will summarize recent advances in new and emerging research models for sepsis, including human-based in vitro systems, highly tailored animal models, AI and digital models analyzing vast datasets to define patient subgroups and predict outcomes, and the FAMOUS framework ensuring that therapies are tested against the specific mechanism they are designed to target. We will discuss the strengths and limitations of these models, reflecting the clinical course of sepsis, and discuss the future directions in this subject area.

## 1. Introduction

Sepsis animal models are crucial tools for studying the mechanisms of sepsis and testing therapeutic strategies before clinical trials. Because sepsis is a complex, systemic inflammatory response to infection, animal models help researchers replicate its immunological, hemodynamic, and metabolic disturbances. Traditionally, the most used animal models for sepsis research are mice because of the ease of experimentation, the availability of genetically engineered species, and the relatively low cost [1,2,3,4,5]. There are several quite commonly used mouse models for sepsis, each with its own advantages and limitations. For example, the cecal ligation and puncture (CLP) model is the most widely used and clinically relevant model of polymicrobial sepsis. The cecum is ligated and punctured (usually once or twice) to allow fecal material to leak into the peritoneum, inducing peritonitis. The advantages are that it mimics human intra-abdominal sepsis well and produces both hyperinflammatory and immunosuppressive phases. The limitations are technically variable (depending on needle size, ligation length, etc.) and require surgical expertise and careful standardization [6]. The lipopolysaccharide (LPS)-induced endotoxemia model uses LPS (endotoxin) injection to trigger systemic inflammation via TLR4 activation to mimic the hyperinflammatory status at the early stage of sepsis. The advantages of this model are that it is easy to perform, reproducible, and inexpensive. The limitations are that it lacks an infectious component (no live bacteria) and represents more of a “cytokine storm” than true infection-associated sepsis. The anti-TNFα therapy showed highly promising results in the LPS-induced sepsis model but failed in human clinical trials, making many investigators question this model [7]. The bacterial injection model involves injecting live bacteria (e.g., *E. coli*, *S. aureus*, *P. aeruginosa*) intravenously, intraperitoneally, or subcutaneously. The advantages of this model are controlled bacterial dose and species, and it is useful for studying host–pathogen interactions. The limitations are that it may not replicate polymicrobial human sepsis, and the route of infection significantly affects outcomes [8]. Another model is colon ascendens stent peritonitis (CASP). A stent is inserted into the ascending colon to allow continuous leakage of feces into the peritoneal cavity. The advantages of this model are producing a persistent infection resembling human peritonitis and allowing study of immune modulation over time. The limitations are technically demanding, and control of infection load is complex [9]. The cecal slurry sepsis model is an injection of murine cecal slurry, which induces acute peritonitis. The advantages are mimicking human intra-abdominal sepsis by introducing a complex, polymicrobial bacterial load; standardized slurry preparation and dosing allow better control over sepsis severity compared to cecal ligation and puncture (CLP); disease severity can be titrated by altering slurry concentration or volume; and it is simple to perform, enabling high-throughput studies and consistent induction across animals. The limitations of this model are that it does not fully replicate the anatomical and immunological dynamics of gut perforation, and microbial composition can vary between slurry preparations unless rigorously standardized [5].

When choosing a model, researchers typically weigh clinical relevance (infection type, pathogen, host response), reproducibility and technical ease, cost and ethical factors, and study goals (e.g., immune response vs. antibiotic efficacy). However, there is no animal model that can fully replicate human sepsis, which causes debate about whether we need to maintain or abandon these animal models [10]. Nevertheless, there is an urgent need to develop new research models or refine current animal models for sepsis research.

## 2. Advanced Human-Based In Vitro Models

Advanced human-based in vitro models have transformed the study of sepsis by using human cells rather than animals. Animal cell lines have been used in many studies of infectious diseases, which have been useful for the development of many antiviral drugs and vaccines for infectious diseases. However, due to discrepancies between the properties of these cell lines and those of in vivo human cells, it is difficult to simulate the host response and tissue damage caused by pathogens using these methods [11]. In fact, approximately 90% of drugs passing preclinical evaluations fail in clinical trials due to inaccurate human response predictions [12]. That is why using in vitro models stands out as a potential way to accurately simulate the pathophysiology of patients and fill the translation gap from animal models to human disease. One method that has been gaining traction is organ-on-a-chip (OOC) technology, which are microfluidic devices that line human cells to mimic the structure and function of human organs. The device is connected to a peristaltic pump to perfuse medium and a vacuum pump to stretch cells, and multiple cell types can be arranged in a 3D manner to mimic internal organ structures [11]. This in turn allows for the evaluation of cellular responses, cell–cell interactions, and organ structure disruptions.

Specific uses of OOCs include introducing pathogens (*E. coli* or *S. aureus*) into the microfluidic “bloodstream” channel to observe real-time human immune responses, endothelial barrier breakdown, and coagulation. Two or more OOCs, like the lung-chip, liver-chip, or kidney-chip, can be connected with a common fluidic circuit to study organ crosstalk and whole-body physiology [13] during sepsis or other infectious diseases, which is a key feature impossible to study in a single cell type dish in animal cell models. Multi-organ chips connect several organ systems through shared perfusion circuits, mimicking blood circulation. This comes from the arteriovenous (AV) mixing reservoir that is integrated into the system to provide mixing of medium, mimicking blood mixing in central circulation. From this, we can sample fluids that are analogous to peripheral blood in patients [13]. Functionally, these chips can cycle signals like cytokines and metabolic byproducts from one organ to another and mix throughout the system, effectively mimicking organ crosstalk. The advantages of this model system include enabling the study of how local infection in one organ can lead to whole-body infection, a key feature of sepsis. OOC technology is very applicable for sepsis due to its ability to culture different tissues together and capture different aspects of sepsis pathophysiology. A prime example of this is when researchers mimicked sepsis onset from the lungs, where they studied the correlation between inflammation and coagulation by delving into the interaction between the human primary alveolar epithelium and endothelium, blood, and infectious signals using real-time imaging of thrombosis [14].

Another unique application of the OOCs is that they can be personalized to reflect individual physiology by including blood samples, cells from specific donors (e.g., an elderly patient vs. a young one), or induced pluripotent stem cells (iPSCs) [15]. By creating and combining multi-organ and patient-specific OOC models from iPSCs, the genetic background of disease can be studied at the systemic level. It is important to study genetic variation within sepsis because both host genetics and pathogen characteristics determine the outcome of sepsis patients. In fact, OOCs may be ideal for investigating both local and systemic effects of crucial genes within sepsis [14].

The intestine plays a key role in pathophysiological events that lead to sepsis within a multi-organ system. The villi in the intestinal barrier are compromised in sepsis, and organoids have stood out to model this behavior in vitro. Intestinal organoids are 3D self-organizing epithelial structures in vitro, made up of intestinal stem cells and epithelial cells [16]. In a study by Huang et al. [16], LPS was used to induce intestinal injury resembling sepsis, and in vitro organoids were used to model the damage. They found that the in vitro model had highly similar biological characteristics to the intestinal injury model induced by LPS in vivo, suggesting that the intestinal organoids may be useful for studying how sepsis disrupts the gut barrier, a key event in multi-organ failure. In addition, the organoids may provide a more effective way to screen therapeutic drugs and research sepsis intestinal injury in comparison to the typical animal models.

Ex vivo perfused organs, explanted organs that are kept alive in a chamber with a perfusate, are another viable option to test therapies for sepsis in a complex, human tissue environment without any risk to a patient. One example of this is ex vivo lung perfusion (EVLP), which can be used to assess lung function and treat injuries. Furthermore, there is no risk of damaging other organs from treatment, making EVLP a suitable candidate to study broad-spectrum antibiotic therapy. The system allows researchers to analyze inflammatory cytokines and chemokines released into the perfusate, essential biomarkers of tissue injury in sepsis research [17]. In a study by Nakajima et al. [17], explanted lungs were infected with *Escherichia coli* and *Pseudomonas aeruginosa* to evaluate antibiotic therapy under EVLP. They found that when broad-spectrum antibiotic therapy during EVLP reduced endotoxin levels, which are highly associated with cytokines and chemokines, it suggested that the antibiotic therapy likely reduced the production of these early response inflammatory mediators. These results highlight the application of EVLPs, as we can test antibiotic therapies that modulate inflammatory mediator levels, an essential component of sepsis treatment development, creating a specific mechanistic way of testing treatments that traditional sepsis models lack.

Despite their promise, advanced human-based in vitro models such as organ-on-a-chip (OOC) systems, organoids, and ex vivo perfused organs face several limitations and technical challenges. OOC platforms require complex microfluidic setups, including precise control of perfusion, mechanical forces, and multi-cellular organization, which can reduce reproducibility and limit scalability for high-throughput drug screening. Standardization across laboratories remains challenging, as variations in chip design, flow dynamics, and cell sources can lead to inconsistent results. Additionally, while multi-organ chips mimic organ crosstalk, they still lack full systemic components such as neural, endocrine, and adaptive immune system interactions, constraining their ability to fully recapitulate whole-body sepsis dynamics.

Patient-specific OOCs and iPSC-derived models introduce further challenges related to differentiation efficiency, cellular maturity, and long culture times, which may not fully reflect adult tissue physiology. Organoids, while effective at modeling localized tissue responses, often lack vascularization, immune cell integration, and mechanical forces, limiting their ability to simulate systemic inflammatory responses seen in sepsis. Ex vivo perfused organ systems are constrained by limited organ availability, short experimental time windows, and the absence of long-term systemic regulation, making them unsuitable for chronic studies. Collectively, while these human-relevant models significantly improve translational potential compared to animal models, technical complexity, cost, and incomplete systemic representation remain key barriers to their widespread adoption in sepsis research.

## 3. Genetically Defined and Humanized Animal Models

Traditional animal models face many challenges in creating effective therapies for sepsis. However, genetically defined and humanized animal models, specifically humanized mice, are far more sophisticated than traditional ones, which have specific genetic modifications to reflect human vulnerabilities. Traditional mouse models have unsuccessful translation of therapies to humans for sepsis because they have a significantly higher resistance to sepsis than humans, and mice’s immune systems have many distinctive features that cannot be controlled [18]. Humanized mice emerged as a solution to this problem by reflecting the human immune system rather than their own. These mice are xenotransplant immunodeficient animals that are engrafted with human immune cells or stem cells, mimicking the human immune system with higher accuracy and inactivating the native mouse immune system. Many humanized mouse models are especially useful in the study of how our immune system will respond to infection. A review by Laudanski [18] has highlighted how these models prove advantageous by describing the mechanism of apoptosis, interactions of staphylococcal superantigens with the immune system, development of vasculitis in hemorrhagic fever and meningitis, and short- and long-term reprogramming of the leukocyte via epigenetic mechanisms in ways like that of the human immune system. Collectively, these advantages suggest that humanized mice not only replicate the complexity of the human immune system’s response to infection but also serve as a tool for preclinical trials of therapies for sepsis that traditional animal models could not reflect.

Despite improvements over traditional animal models, genetically modified, humanized, and frailty-based mouse models retain important limitations in sepsis research. Humanized mice, while better at recapitulating aspects of the human immune response, often exhibit incomplete or imbalanced immune system reconstitution, as not all human immune cell lineages engraft or function equivalently. These models frequently lack proper human cytokine signaling, lymphoid architecture, and immune cell maturation, which can distort immune responses and limit their predictive value for therapeutic outcomes. Additionally, the immunodeficient background required for xenotransplantation increases susceptibility to infections unrelated to sepsis and complicates experimental control. Humanized mouse models are also costly, technically demanding, and exhibit significant inter-animal variability due to differences in efficiency in engraftment.

Older adults are known to be more vulnerable to sepsis. However, preclinical research on sepsis is typically conducted using traditional lab mice that are young and healthy. This results in decreased validity because age is an independent predictor of poor outcomes of sepsis. Thus, frailty models that use aged mice appear to stand out to bypass these fallbacks [19]. In a study by Sharma et al. [19], a fecal-induced peritonitis (FIP) mouse model was tested to explore the impact of aging in response to sepsis. The results were impactful because in the 12 h study, the aged FIP mice (89%) demonstrated increased inflammation and lung injury compared to the young FIP mice (42%). Furthermore, the aged FIP non-survivors exhibited elevated IL-6, TAT, CFDNA, and CCL2 and decreased IL-10, and impaired bacterial clearance compared to young FIP non-survivors. These findings by Sharma et al. [19] are significant because they demonstrate how age and frailty greatly affect how the body responds to sepsis. The elevated cytokines and reduced anti-inflammatory control (low IL-10) of the aged mice reflect the immune response typical of sepsis in older, frail adults. By capturing a more accurate response to sepsis, frail and aged mouse models can provide an increased validity of preclinical research and are useful in identifying specific age-related mechanisms in sepsis as well as age-specific therapies [19]. Similarly, a study by Walston et al. [20] highlighted how increased inflammatory mediators and weaknesses were one of the most common characteristics of human frailty in older adults. This was reflected in their experiment as aging mice developed decreased strength and increased inflammation, consistent with two critical components of human frailty. This imbalance mirrors the results of the aged FIP mice from the study by Sharma et al. [19], validating the use of frailty-modeled mice over the traditional younger mice in producing effective therapies for sepsis that target age-specific mechanisms.

Frailty and aged mouse models introduce further challenges related to experimental complexity and resource demands. Aged mice are expensive to maintain, have higher baseline morbidity, and display heterogeneous health statuses that complicate data interpretation and reduce reproducibility. Moreover, murine aging does not fully capture the multifactorial nature of human frailty, which involves comorbidities, polypharmacy, and long-term environmental exposures. While aged mice are a better model of inflammatory dysregulation seen in older adults with sepsis, species-specific differences in immune signaling, metabolism, and organ reserve remain significant barriers to translation. Collectively, although advanced animal models improve biological relevance compared to young, healthy mice, persistent interspecies differences, high costs, and technical variability continue to limit their ability to fully predict human responses and therapeutic efficacy in sepsis.

## 4. Digital and AI-Driven Models (In Silico)

We can utilize digital and AI-driven models that leverage large datasets to enhance our understanding and prediction of sepsis with greater efficacy than the current methods. These “in silico” models can simulate disease onset, predict therapy responses, and enable virtual testing of interventions with little risk to the patient. Sepsis consists of many different individual pathophysiologies and treatment responses. Traditional sepsis trials do not consider the heterogeneity of sepsis and employ a one-size-fits-all approach when managing critically ill patients with sepsis, highlighting the need for individualized treatment strategies for sepsis [21]. However, instead of pre-defining groups, machine learning algorithms (such as clustering algorithms) are applied to large patient datasets (including vitals, labs, and genomics) to discover distinct subclasses of sepsis patients (endotypes).

A study by Mekata et al. [22] applied a clustering algorithm to identify distinct endotypes, then investigated the association between those endotypes and mortality risks. They identified three different endotypes: coagulopathic, inflammatory, and adaptive. The coagulative exhibited upregulated coagulation signaling, and the adaptive demonstrated enhanced immune cell responses and elevated T and B cell compositions. The inflammatory endotype exhibited upregulated TNF-α/NF-κB signaling and IL-6/JAK/STAT3 pathways, accompanied by an increased neutrophil composition. Importantly, they were able to identify different mortality rates of each of the endotypes, with coagulopathic individuals having a significantly higher risk of mortality than those of the adaptive endotype. The impact of this is tremendous, as this endotype-based approach can help develop more precise therapeutic strategies for sepsis by providing a framework to understand the wide biological mechanisms that underlie sepsis. Investigating the way certain endotypes respond to treatment can help us deduce what strategy we need to utilize specific to the patient’s needs, increasing our capacity for individualized sepsis treatment.

Using a digital “twin” of a human population, built from real-world data, researchers can simulate a clinical trial. This allows them to test a proposed therapy in the virtual population, predict its likely success, identify the optimal patient population, and determine the optimal dosing before investing millions in a real trial. A study by Lal et al. [23] developed a digital “twin” patient model using agent-based modeling and simulation (ABMS), discrete-event simulation, and a Bayesian network model to simulate the patient and the environment across multiple stages in response to treatment during the first 24 h of sepsis. For example, the ABMS model of sepsis was used to simulate the action and interactions of the cardiovascular, neurologic, renal, respiratory, gastrointestinal, inflammatory, and hematology systems. The model considered the patient’s case timeline, actionable clinical markers, and graduated clinical interventions, allowing it to generate more accurate future predictions. This model for critically ill patients has a wide application for clinical interventions, as it can be tested in a virtual environment, minimizing the risk to real patients. By considering specific clinical markers that impact the interactions between different organ systems, researchers can fill in important knowledge gaps [23]. By being a predictive model, digital “twin” models can serve not only as a research tool but also as an educational model that can discover new mechanisms at play during sepsis, thereby increasing the effectiveness of current therapies.

Despite their strong potential, digital and AI-driven in silico models face significant limitations that constrain their clinical translation in sepsis research. The performance and validity of machine learning models are highly dependent on the quality, completeness, and representativeness of the datasets. Clinical sepsis data are often heterogeneous, noisy, biased toward specific institutions or populations, and affected by missing or inconsistent measurements, which can lead to biased endotype discovery and limited generalizability. Additionally, clustering-based endotypes may be sensitive to algorithm choice, feature selection, and preprocessing methods, resulting in unstable or non-reproducible classifications across cohorts.

Interpretability remains a major challenge for complex AI models, as many machine learning approaches function as “black boxes,” limiting mechanistic insight and clinician trust. While digital twin and agent-based models can simulate multi-organ interactions, they rely on simplifying assumptions and incomplete biological knowledge, which may overlook emergent or nonlinear dynamics of sepsis. Model validation is also difficult, as virtual trial predictions must be rigorously benchmarked against prospective clinical outcomes, yet real-world validation datasets are scarce. Furthermore, integrating AI-driven predictions into real-time clinical workflows presents technical, regulatory, and ethical challenges, including computational demands, data privacy concerns, and the risk of algorithmic bias influencing treatment decisions. Consequently, while silico and digital twin models offer powerful tools for hypothesis generation and trial optimization, they currently complement rather than replace experimental and clinical sepsis research.

## 5. The Framework of FAMOUS: Focusing on Mechanisms

A pivotal shift in sepsis research is the FAMOUS framework (Focus on Assessing Mechanism of Disease and Using Stratification). This is not a model itself but a new approach to using models. Rather than just testing a drug in a generic sepsis model, this approach highlights identifying a specific mechanism with an appropriate model and understanding the reasons for the results. The current sepsis model focuses on generic treatments and seeing if they improve survival. However, this is largely inefficient because they do not consider the different underlying pathophysiology that exists in each patient. There is not enough understanding of the mechanisms surrounding sepsis, as it is not homozygous in nature. For future success, we need to adopt a new approach that tries to understand illness, integrates basic and clinical science, and creates specific models that foster individualized treatment [24].

To move past the one-size-fits-all approach, the FAMOUS framework helps test a therapy against a specific, rather than a generic, mechanism it is designed to target. The first step is to identify a specific mechanism that the therapy is designed to target. This can be something like immune paralysis via low HLA-DR. Then, selecting a specific model that replicates that specific mechanism, like a humanized mouse model or a blood sample from an immunosuppressed sepsis patient. The next step is to stratify by testing potential therapies only in subjects (animal or human) who exhibit that mechanism. Lastly, precise biomarkers, including inflammatory cytokine levels (TNF, IL-1, and IL-6, etc.), organ injury parameters (AST, ALT, CK, and LDH), and immune paralysis (HLA-DR, etc.), should be used to measure whether the drug successfully modulates the intended target, rather than relying on general measures such as survival. By considering underlying mechanisms, specific models and patients, and biomarkers, the FAMOUS framework serves to integrate basic and clinical science to produce targeted, patient-specific therapies that overcome the failures of generic sepsis models.

While the FAMOUS framework represents an important paradigm shift toward mechanism-based and stratified sepsis research, its implementation faces several practical and technical challenges. A major limitation is the incomplete understanding of sepsis mechanisms themselves; many proposed pathways, such as immune paralysis or hyperinflammation, overlap temporally and spatially within individual patients, complicating clear mechanistic classification. Reliable identification of patient subgroups, therefore, depends on the availability of robust, validated biomarkers, which are currently limited, inconsistently measured, or not routinely available in clinical settings.

Model selection under the FAMOUS framework also presents challenges, as no single experimental model fully recapitulates isolated sepsis mechanisms without introducing confounding pathways. Humanized animal models, patient-derived samples, and advanced in vitro systems may capture specific features of disease, but each has inherent technical limitations, variability, and scalability constraints. Furthermore, stratifying patients or experimental subjects by mechanism reduces sample sizes, increasing statistical complexity and potentially limiting the power of preclinical and clinical studies. Finally, translating mechanism-specific findings into clinical practice requires changes in trial design, regulatory frameworks, and clinician workflows, which may slow adoption. Despite these challenges, the FAMOUS framework offers a more rational and biologically grounded approach to sepsis research, provided these methodological and infrastructural barriers can be addressed.

## 6. Future Directions

The current new research models for sepsis have been summarized in Table 1. The future of sepsis research models is moving away from simplistic and one-size-fits-all approaches toward highly complex, dynamic, and personalized systems that mirror the profound heterogeneity of the human condition. Conceptually, future models must mimic specific human endotypes (e.g., hyperinflammatory vs. immunosuppressive) identified from clinical data, rather than testing therapies on a generic “septic” model. In addition to modeling personalized “Host”, future models need to incorporate genetic diversity (e.g., using mice from the collaborative cross or diversity outbred populations) and comorbid conditions (age, diabetes, cancer) to understand how host genetics shape responses. Technically, multi-organ chips will be connected by linking endothelial-lined microvessels with modules representing liver, kidney, lung, and gut to study organ-organ crosstalk and systemic inflammation dynamics in a controlled, human-relevant context. The system will further incorporate patient-derived immune cells (macrophages, neutrophils) to create a more complete “immune-on-a-chip” and integrate living human microbiome communities with gut epithelial models to study the gut-lung axis and bacterial translocation. The key challenges for the future models are: (1) standardization vs. relevance: how to standardize these incredibly complex models for reproducible research without losing clinical relevance; (2) cost and accessibility: advanced organ-chips and humanized mice are expensive and technically demanding; (3) data integration: creating frameworks to integrate data across scales—from molecular (in vitro) to organismal (animal) to digital (AI); and (4) the new models need to be validated.

In summary, there is common sense that more clinically relevant models with higher translational value are urgently needed for sepsis research. AI-assisted new technology helps to integrate big data and provide model systems to distinguish different endotypes and design personalized therapeutic plans. Multi-organ-on-chips need to be further standardized and tested in their translational value. Organoids from iPSCs are promising to establish future personalized and targeted therapies for sepsis patients.

## Figures and Tables

**Table 1:** Comparison of new sepsis research models: applications, strengths, and limitations.

Approach	Primary Applications	Key Strengths	Major Limitations
Advanced Human-Based In Vitro Models (Organ-on-a-chip, organoids, ex vivo perfusion)	Human-specific immune and tissue responses Organ and multi-organ sepsis mechanisms Drug screening and personalized modeling	High human relevance Real-time monitoring of cell–cell and organ crosstalk Patient-specific and genetic modeling possible	Technically complex and costly Limited scalability and standardization Incomplete systemic physiology
Humanized Animal Models	In vivo study of human immune responses Preclinical testing of immunotherapies	Recapitulate key aspects of human immunity Enable mechanistic immune studies not possible in vitro	Incomplete immune reconstitution Altered cytokine signaling and immune maturation High cost and inter-animal variability
Frailty and Aged Animal Models	Age-related sepsis susceptibility Identification of age-specific mechanisms and therapies	Better reflect sepsis biology in older adults Capture inflammatory imbalance and immune dysfunction	Expensive High variability and reduced reproducibility Murine aging incompletely models human frailty
Digital and AI-Driven Models (In Silico)	Endotype discovery and risk stratification Outcome prediction and virtual trials	Capture sepsis heterogeneity Low risk, cost-efficient, scalable Enable precision medicine strategies	Dependent on data quality and bias Limited interpretability Challenging clinical validation
FAMOUS Framework (Mechanism-based stratification)	Guide model selection and trial design Mechanism-targeted therapy development	Moves beyond one-size-fits-all approaches Integrates basic and clinical science Supports biomarker-driven precision therapy	Incomplete understanding of sepsis mechanisms Limited validated biomarkers Smaller stratified cohorts reduce statistical power

## Data Availability

No new data were created or analyzed in this study.

## Abbreviations

CLP	cecum ligation and puncture
LPS	lipopolysaccharide
FAMOUS	Focus on Assessing Mechanism of Disease and Using Stratification
ABMS	agent-based modeling and simulation
FIP	fecal-induced peritonitis
EVLP	ex vivo lung perfusion
iPSCs	induced pluripotent stem cells
OOC	organ-on-a-chip
CASP	colon ascendens stent peritonitis
TLR4	toll-like receptor 4
